# A Case of Chronic and Relapsing Paget Disease of the Vulva

**DOI:** 10.1055/s-0039-1687861

**Published:** 2019-04-24

**Authors:** Rita Bouceiro-Mendes, Maria Mendonça-Sanches, Luís Soares-de-Almeida, Isabel Correia-Fonseca

**Affiliations:** 1 Department of Dermatovenereology, Centro Hospitalar Universitário Lisboa Norte, Hospital de Santa Maria, Lisboa, Portugal; 2 Institute of Molecular Medicine, Faculdade de Medicina de Lisboa, Lisboa, Portugal

**Keywords:** extramammary Paget disease, Paget disease of the vulva, total vulvectomy, radiotherapy

## Abstract

Extramammary Paget disease is a rare neoplastic condition that more commonly affects postmenopausal Caucasian women. Although the vulvar area is the most frequently affected location, it corresponds solely to 1 to 2% of all vulvar malignancies. A 72-year-old female patient was observed in our outpatient clinic with a 2-year history of an erythematous and pruritic plaque on the vulva. Histopathology and immunohistochemistry studies were compatible with extramammary Paget disease of the vulva. Associated neoplastic conditions were excluded. Due to multiple relapses, the patient was submitted to three surgical interventions, including a total vulvectomy, and to external radiotherapy. The present case illustrates the chronic and recurrent nature of extramammary Paget disease despite aggressive procedures as well as the challenge in obtaining tumor-free resection margins.

## Introduction

Extramammary Paget disease (PD) is a rare entity,^1^ ^2^ ^3^ ^4^ accounting for only 6% of all PD. It is a neoplasm that frequently presents in areas where apocrine glands are abundant, such as the anogenital region and, less frequently, the axillae.^2^ ^3^ ^5^

Its prevalence is unknown, but it is more common in Caucasian and postmenopausal women,^1^ ^3^ with the vulvar area being the most frequently involved location.^2^ ^3^ ^5^ Differential diagnosis with infectious or inflammatory diseases is difficult, and a histological study of all the suspected lesions is essential.^3^ ^6^ ^7^ ^8^

Surgery, with total excision, remains the treatment of choice, although other therapeutic approaches such as radiotherapy, photodynamic therapy, CO_2_ ablative laser therapy, interferon alpha, topical 5-fluorouracil (5-FU) and topical 5% imiquimod have been used with variable success.^2^ ^3^ ^4^ ^5^ ^6^ ^9^ Regardless of the adopted therapy, the disease seems to follow a chronic course with high relapsing rates, ranging from 15 to 72%.^2^ ^4^ ^5^ ^7^

## Case Report

**Fig. 1:**
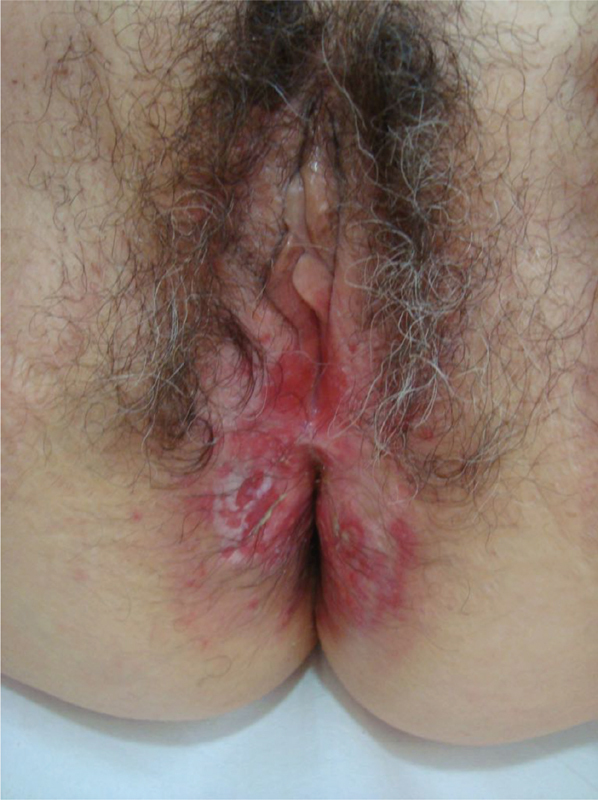
Clinical presentation: erythematous plaque covered by fine and whitish scales, with some areas of erosion.

An otherwise healthy 72-year-old postmenopausal Caucasian woman was seen in our outpatient clinic due to a 2-year history of an intensely itchy vulvar plaque. The patient reported that it all began with a small erythematous and pruritic plaque on the vulvar region that gradually enlarged over a 2-year period. She had been previously observed in another medical facility and had been treated with topical corticosteroids and cryotherapy, without clinical improvement. The dermatological observation revealed an erythematous plaque in the region of the furcula and in the perianal area, covered by thin and whitish scales, with some areas of erosion (Fig. 1). There were no similar cutaneous lesions elsewhere, nor regional lymphadenopathy. The gynecological evaluation, including rectal examination, was otherwise unremarkable.

**Fig. 2:**
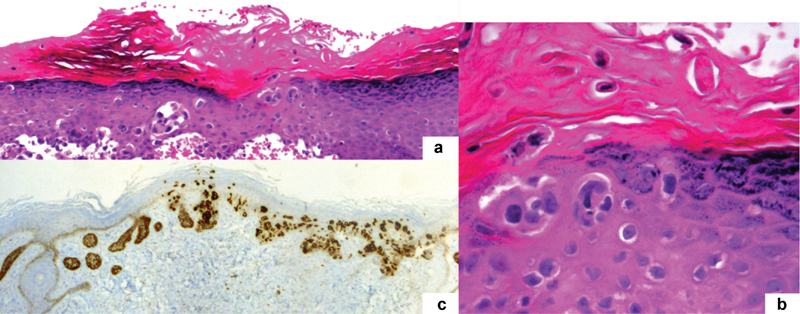
Histopathological and immunohistochemistry features: (a) Pagetoid infiltration of the epidermis by pleomorphic large cells, isolated or in nests, with clear cytoplasm and a prominent nucleolus (hematoxylin and eosin x100); (b) Pagetoid cells (hematoxylin and eosin x400); (c) Positive cytokeratin 7 staining (CK7, x100).

Laboratory investigations, including blood count, carcinoembryonic antigen (CEA), cancer antigen 125 (CA-125) and cancer antigen 15.3 (CA-15.3) were within the normal range. A skin biopsy revealed a pagetoid infiltration of the acanthotic epidermis by pleomorphic large cells, isolated or in nests, with clear cytoplasm and a prominent nucleolus. The mitotic index was low and there was no contiguity with the hair follicle, nor evidence of dermis invasion (Figs. 2a and 2b). These cells were Periodic acid–Schiff (PAS) and Alcian blue positive. Immunohistochemical studies showed immunoreactivity for cytokeratin 7 (CK7) (Fig. 2c), CEA, and gross cystic disease fluid protein (GCDFP-15), and negativity for cytokeratin 20 (CK20), CA-125, estrogen receptors, S100, Melan-A and p63. These findings are consistent with PD. Multiple skin biopsies of the involved and surrounding uninvolved skin were performed (vulvar mapping), with no evidence of dermal invasion. To exclude associated malignancies, mammography, breast ultrasound, cystoscopy, colonoscopy and an abdominal-pelvic computed tomography (CT) were performed, all with unremarkable results.

**Fig. 3:**
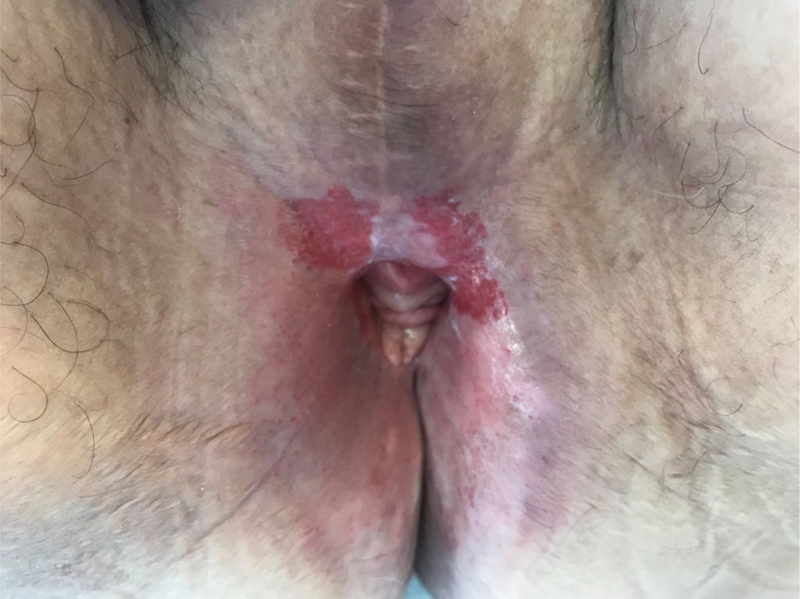
Clinical presentation: Paget disease of the vulva relapse (erythematous plaque) after total vulvectomy and radiotherapy.

The patient underwent local surgical excision of the vulvar lesion, with positive lateral surgical margins. She was kept on close follow-up, with monthly assessments by a multidisciplinary team. Nine months after the procedure, due to recurrence of the disease, a wide surgical excision was performed, revealing once again positive surgical margins. Considering the involved surgical margins, treatment with imiquimod 5% cream was proposed, to be started after complete recovery from the surgical procedure. Unfortunately, 3 months after the latter intervention and before the initiation of the imiquimod treatment, relapse of the disease was documented on a skin biopsy. Given the good performance status of the patient and her will to undergo further surgery, a total vulvectomy with fasciocutaneous flap reconstruction was performed, achieving tumor-free resection margins. The patient remained clinically stable for 4 years, when a new recurrence was documented. After the approval of the patient, external radiotherapy at a total dose of 59.4 Gy was initiated. The patient completed the proposed therapeutic scheme within 2 months, with good tolerance. By the end of the treatment, clinical regression of the lesion was observed. However, within 3 months, a new suspicious lesion was detected (Fig. 3), and a skin biopsy confirmed a new recurrence of PD. Considering the comorbidities and difficulties associated with multiple surgical interventions and frequent relapses, an alternative treatment with topical imiquimod 5% cream applied every other day on the affected area was once again proposed. Due to personal constraints, the patient moved to another medical institution and was lost to follow-up.

## Discussion

Paget disease of the vulva (PDV) is a rare malignancy that should be considered in the differential diagnosis of well-defined, erythematous and scaly plaques in the vulvar region, particularly in Caucasian postmenopausal women.^2^ ^3^ ^5^ ^7^ Skin biopsy of all suspected lesions is mandatory. The etiopathogeny of PDV is not well established. While some studies suggest an intraepidermal origin in adnexal structures, recent theories have postulated a possible origin in Toker cells or in the mammary glands located in the interlabial sulcus.^7^

Paget cells have pale and abundant cytoplasm with large atypical nuclei and prominent nucleoli. These cells contain mucin, staining positive for PAS, colloidal iron, Alcian blue and mucicarmine. Immunohistochemistry is often used to diagnose PD, revealing positivity for CK7 and CEA. They do not express squamous-cell differentiation markers such as p63 (useful in the differential diagnosis with Bowen disease), nor melanocytic markers such as S100, Melan-A and Human Melanoma Black 45 (HMB-45) (for melanoma differential diagnosis).^6^ ^7^ ^10^ It is essential to exclude associated neoplasms, such as genitourinary and gastrointestinal carcinomas, since they are present in ∼ 30% of the cases.^3^ ^5^ ^6^ Immunohistochemistry studies may also be useful in this clinical setting – positivity for CK20, uroplaquine-III and GATA-3 is associated with urothelial cancer, while positivity for CK20, CDX2 and mucin 2 (MUC2) is related to anorectal adenocarcinoma. On the other hand, positivity for GCDFP-15, as presented in the present clinical case, is associated with primary skin disease.^6^ ^7^ ^10^

The classification of PDV, proposed by Wilkinson et al^11^ distinguishes the primary/cutaneous (type 1) from the secondary/non-cutaneous (type 2 and type 3). Type 1 is further divided according to the presence or absence of dermal invasion.^7^ ^11^ To exclude dermal invasion, multiple biopsies of the involved and surrounding uninvolved skin are recommended. In primary PDV, the depth of infiltration is the most important prognostic factor, with a 5-year mortality of ∼ 40% in cases of documented invasive disease.^1^

The treatment for PDV is challenging due to frequent relapses, regardless of the treatment option.^12^ Surgery has been the standard treatment for PDV but, because of its multifocal and subclinical skin involvement, surgical margins are frequently positive. As such, and although the results have not been encouraging (since involved surgical resection margins are neither associated with recurrence rates nor with overall survival), surgery with intraoperative margin control (Mohs microsurgery) is the preferred surgical method.^5^ ^6^ ^9^ ^10^ Nonsurgical therapeutic approaches are still reserved for patients who are not candidates for surgical resection or who decline it.^13^ ^14^ Radiotherapy can also be considered a therapeutic option in these cases and/or in patients with multiple relapses.^15^

Currently, some studies, mainly case reports, have investigated and documented the effects of conservative topical regimens such as 5-fluorouacil (5-FU), imiquimod and bleomycin, which could decrease the comorbidities associated with both surgery and radiotherapy in patients with PD.

5-Fluorouacil has selective toxicity in premalignant and malignant epithelial lesions, as it inhibits thymidylate synthetase, disrupting DNA synthesis in active proliferating cells. Despite its treatment indication in PDV, there are surprisingly few studies evaluating its efficacy as monotherapy. Presently, most case reports demonstrate that it should not be considered as a first-line treatment in extramammary PD due to its erosive/ulcerating effects, which may provide an incorrect impression of lesion resolution, not recognized on biopsy specimens that may show a histologic persistence of the disease.^16^ ^17^ ^18^ ^19^ As such, topical 5-FU has been most commonly used in combination treatment with systemic chemotherapy in advanced cases of extramammary PD or in combination with other topical therapies in cases of resistant disease.^20^ ^21^ ^22^ It can also be useful prior to surgery to delineate the extent of cutaneous involvement due to its inflammatory effects.^18^ ^19^

Topical 5% imiquimod cream was first reported as a treatment for PDV in 2002.^23^ Since then, multiple case reports, a few retrospective studies, and a recent prospective pilot trial of topical imiquimod 5% cream in women with PDV have demonstrated its efficacy (after exclusion of underlying or coexistent malignancy). Reported side effects are usually mild and include pain, irritation, erythema, erosion and residual hypopigmentation.^8^ ^17^ ^24^ ^25^ ^26^ ^27^ ^28^ Imiquimod is an immunomodulatory agent targeting the toll-like-receptor 7 as a receptor agonist and it has direct antitumor activity.^7^ ^17^ ^25^ In 2014, a retrospective study by Luyten et al^27^ involving 21 cases treated with imiquimod reported a 52.4% complete response rate, a 28.6% partial response rate, and no documented cases of disease progression. In the same year, Marchitelli et al^25^ presented a case series of 10 patients reporting similar results. Machida et al^28^ conducted a systematic review of the literature and found an overall 73% complete response rate and a 7% persistent disease rate with imiquimod treatment. More recently, in 2017, Dogan et al^8^ conducted another systematic review on the therapeutic efficacy of imiquimod. Pooled rates of complete remission and of partial remission of 71% and of 16%, respectively, have been reported.^8^ According to these studies, topical 5% imiquimod cream has been considered a safe and feasible option for PDV, especially for patients suffering from recurrent PDV or undergoing multiple surgical resections, or who are poor surgical candidates, being regarded as a viable alternative to surgery. The treatment schedules described in the literature differ widely, ranging from daily to 3 applications per week, with variable durations. It is not yet possible to recommend a dose and duration of imiquimod therapy for PDV. To address this issue, the Paget Trial, a multicenter observational cohort study that includes eight tertiary referral hospitals in Netherlands, is currently ongoing. It aims to investigate the efficacy and safety of 5% imiquimod cream in primary PDV using a standardized treatment schedule.^29^

Regarding bleomycin as a topical therapeutic option in PDV, there are scarce case reports and minimal clinical investigations, although it has shown efficacy in the treatment of other skin neoplasms.^4^ ^7^

The present case illustrates the chronic-relapsing course of PDV. Although aggressive surgical therapies and rescue radiotherapy were applied, and regardless of the status of the surgical margins, disease recurrence was a major issue.^2^ ^5^ ^7^ ^8^ Consequently, close follow-up over a long period of time is warranted. The current scarcity of randomized clinical trials and the absence of standardized guidelines^8^ ^9^ make therapeutic approaches a real challenge. A careful and individualized patient evaluation by a multidisciplinary team is mandatory to achieve disease control without overtreatment. Considering the chronic and relapsing course and the high morbidity associated to radical and repeated vulvar surgery, we highlight the role of noninvasive therapies as an alternative treatment in selected cases, especially the use of topical 5% imiquimod cream as an option for women with involved resection margins after surgery or with disease recurrence.

## Conclusion

Regardless of the adopted therapy, PDV seems to follow a chronic course with high relapse rates. The challenge in dealing with PD is to achieve disease control without overtreatment. Surgery remains the standard of care, but noninvasive therapies are emerging and should be considered in selected cases.
